# Cardiomyocyte Atrophy, an Underestimated Contributor in Doxorubicin-Induced Cardiotoxicity

**DOI:** 10.3389/fcvm.2022.812578

**Published:** 2022-02-25

**Authors:** De-Shu Chen, Jing Yan, Ping-Zhen Yang

**Affiliations:** ^1^Department of Cardiology, Laboratory of Heart Center, Zhujiang Hospital, Southern Medical University, Guangzhou, China; ^2^Heart Center of Zhujiang Hospital, Guangdong Provincial Biomedical Engineering Technology Research Center for Cardiovascular Disease, Guangzhou, China; ^3^Heart Center of Zhujiang Hospital, Sino-Japanese Cooperation Platform for Translational Research in Heart Failure, Guangzhou, China

**Keywords:** doxorubicin, cardiotoxicity, cardiomyocyte atrophy, left ventricular mass loss, cell death

## Abstract

Left ventricular (LV) mass loss is prevalent in doxorubicin (DOX)-induced cardiotoxicity and is responsible for the progressive decline of cardiac function. Comparing with the well-studied role of cell death, the part of cardiomyocyte atrophy (CMA) playing in the LV mass loss is underestimated and the knowledge of the underlying mechanism is still limited. In this review, we summarized the recent advances in the DOX-induced CMA. We found that the CMA caused by DOX is associated with the upregulation of FOXOs and “atrogenes,” the activation of transient receptor potential canonical 3-NADPH oxidase 2 (TRPC3-Nox2) axis, and the suppression of IGF-1-PI3K signaling pathway. The imbalance of anabolic and catabolic process may be the common final pathway of these mechanisms. At last, we provided some strategies that have been demonstrated to alleviate the DOX-induced CMA in animal models.

## Introduction

Doxorubicin (DOX), the most prescribed anthracycline chemotherapy agent, remains one of the most commonly used anti-cancer drugs among the world while its clinical application is limited by its cumulative dose-dependent cardiotoxicity (1, 2). The congestive heart failure (CHF) is the end stage of DOX-induced cardiotoxicity (DIC) and predicts poor prognosis. The incidence of DOX-related CHF reaches to 26% in patients received DOX at a cumulative dose of 550 mg/m^2^ (3). The health of patients with cancer and cancer survivors is threatened by the DIC, unfortunately, the number of both is large. For cancer survivors only, it was reported that there are more than 16.9 million cancer survivors until January 1, 2019 in the United States; this number is estimated to reach more than 22.1 million in the next decade based on the growth and aging of the population alone (4). Nowadays, several strategies are recommended for patients planning to receive high-dose anthracyclines to prevent DIC, such as the use of dexrazoxane or liposomal formulation of doxorubicin, continuous doxorubicin infusion (evidence based; strength of recommendation: moderate) (5). However, there is still lack of evidence to confirm whether these strategies are safe and effective for all patients with cancer receiving chemotherapy (5–7). Although small clinical trials have revealed that conventional drug of heart failure therapy may be beneficial against DIC (8, 9). Cardinale et al. reported that with conventional heart failure therapy, only 11% patients showed complete recovery from DIC in a heterogeneous cohort study of 2,625 patients (10). Therefore, it is vital to uncover the key mechanism of DIC and find a new approach to prevent it.

Several studies have revealed that anthracycline-based chemotherapy (ANbC) accounts for the left ventricular (LV) mass loss in patients with cancer and cancer survivors. In patients receiving ANbC, the LV mass decrease was detected as early as 1 month after the initiation of the therapy (11) and a 5% reduction in LV mass in 6 month was found based on the cardiovascular magnetic resonance detection (12). In the studies focusing on the pediatric and adult cancer survivors, the LV mass reduction still exists more than 20 years after the ANbC therapy (13–17). The severity of LV mass loss is correlative with the cumulative dose of DOX (14, 18). Further, there may not be a safe dose of DOX to avoid cardiotoxicity for that the cardiac abnormalities, such as significantly reduced LV mass and dimension was found in patients who received as low as 45 mg/m^2^ cumulative dose (18).

Although there are multi-factors that can be resulted into the LV mass loss, for example, cancer-associated cachexia like food intake reduction and excess catabolism (19), heart load alteration (20), denervation (21), and bed rest (22). Jordan et al. found that the reduction of LV mass is not necessarily accompanied with the decline of body weight and the heart failure (HF) symptom is not associated with the body weight decrease in patients who received ANbC, indicating a process other than cancer-associated cachexia leads to LV mass loss (12). Consistent finding was reported in animal models, DOX itself caused the heart weight loss in healthy mice and the heart weight (HW)/body weight (BW) index decreases in a dose-dependent fashion of DOX treatment, implicating the possibility that the HW loss is out of portion of BW loss and is caused by the chemotherapy (11). Intriguingly, Pietzsch et al. reported that the cardiac dysfunction induced by cancer alone would nearly recover to the base line, while tumor-bearing mice with DOX treatment showed lower survival rate in the acute phase and long-lasting damage in the gene expression system (23).

The LV mass loss is correlative to the decline of life quality (12) and the increase of major adverse cardiovascular events, such as cardiovascular death, implantable cardioverter-defibrillator therapy, and decompensated heart failure (14). Generally, the LV afterload decreases, the LV mass reduces (20). However, a high afterload was paradoxically found in ANbC-treated patient (12). The same phenomenon was found in animal models. Matsumura et al. found that DOX caused the cardiac atrophy and induced higher blood pressure after angiotensin II treatment (24). In addition, DOX-treated juvenile mice failed to develop cardiac hypertrophic response to late-onset hypertension induced by angiotensin II, which resulted into higher blood pressure, cardiac output decline, and overt mortality (24). Maayah et al. also reported that DOX treatment led to the impairment of the adaptive hypertrophic response to hypertrophic stimuli (25). Insufficient ventricular mass plus high chronic afterload contributes to the progressive contractile deficit, decreased cardiac output, and the establishment of cardiomyopathy (18). These may explain why hypertension markedly increased the risk for coronary artery disease, HF, valvular disease, and arrhythmia in aging adult survivors of childhood cancer (26).

The LV mass loss derives from both cell death (27, 28) and cardiomyocyte atrophy (CMA) (11), leads to cardiac atrophy. It should be noted that cardiac atrophy is different from CMA. The term of cardiac atrophy generally defined as an acquired reduction in the size and mass of the heart (29), is usually evaluated by HW, HW/BW ratio, or HW/tibia length (TL) ratio in animal DIC model. A great number of studies have revealed that DOX caused cardiac atrophy, as indicated by the decrease of HW, HW/TL ratio or HW/BW ratio (30–35). However, several studies reported that DOX caused a reduction of HW and BW, did not affect or increase the HW/BW ratio (36–38). It was reported that the delivery of DOX through intraperitoneal route resulted into peritoneal damage, which interfered the food intake and absorption and caused BW loss (39). Therefore, the preserved or increased HW/BW ratio may originate from the greater BW reduction. Therefore, it may be more appropriate to evaluate the cardiac atrophy by HW/TL ratio or HW alone, which is more evident mostly.

Despite numerous studies focusing on the cell death, less attention was paid on the CMA in DIC studies. However, the weight of cardiomyocyte apoptosis in DIC might be overstated (40). Several studies demonstrated that the contribution of cardiomyocyte apoptosis is low in acute DIC model. Willis et al. reported that CMA rather than cell death determines the cardiac atrophy in acute DIC mice model. They sacrificed mice 7 days after injected with DOX (20 mg/kg) and found that there were barely no increase of serum Troponin-I level and TUNEL-stained cell number in DOX treated mice, however, a 44% reduction of cell cross-section area and an obvious cardiac atrophy were detected (11). Little doxorubicin-induced apoptotic effect in acute DIC model was reported by other groups (41–44). However, it was also reported that DOX caused a great amount of apoptotic cardiomyocyte in an acute DIC model (45–47). Maybe apoptosis plays less important role in cardiac atrophy of acute DIC than we thought. While in a chronic DIC model, cardiomyocyte may undergo a hypertrophy response in a compensated manner (48), CMA was also found in a chronic DIC model (49–51). The controversial results will require further research to clarify, and the role of CMA in the DIC model should be evaluated. In a study including 27 women with breast cancer, patients received the cardiac magnetic resonance image exam at 351–700 days after anthracycline therapy (240 mg/m^2^). Ferreira et al. found that the LV mass index in these patients is correlated with intracellular water lifetime (τ ic; a cardiomyocyte size maker) other than with extracellular volume (ECV), indicating that the cardiac atrophy originates from CMA (52). Cell size shrinkage alone accounted for an ~44% reduction in LV mass, while the increased ECV may attenuate the LV mass loss (52). Except for apoptosis, other forms of cell death had been found and demonstrated to participate in DIC (27), the relative contribution of cell death and CMA in DOX-induced cardiac atrophy needs further studies to illustrate.

Here, we aim to emphasize the importance of CMA in cardiac atrophy, summarize the current knowledge of the effect of DOX on CMA, and provide insight into the underlying molecular mechanism of it, finally discuss some approaches that have been identified to protect it.

## Molecular Mechanism

### Forkhead Box O1 (FOXO1)

Forkhead box O (FOXO) proteins are transcription factors regulating multi physiological and pathological processes included in cardiovascular system. The family contains four members in human, FOXO1, FOXO3, FOXO4, and FOXO6 (53). FOXOs are key regulators in maintaining the muscle mass (54). Depletion of FOXOs has been reported to prevent the muscle loss and weakness through suppressing autophagy–lysosome systems (ALS) and ubiquitin–proteasome systems (UPS) *via* inhibiting the AKT activity (55). Sengupta et al. reported that FOXOs activation may reduce the cardiomyocyte size by promoting autophagy (56). Additionally, Skurk et al. (57) reported that AKT-FOXO3a signaling regulates cardiomyocyte cell size against hypertrophy *via* mediating the expression of atrophy-related genes “atrogenes”. Actually, FOXOs regulates half of the atrogenes by binding their promoters, such as muscle RING finger 1 (MuRF1), muscle atrophy gene-1 (atrogin-1/MAFbx), and Bcl-2 19-kDa interacting protein 3 (Bnip3) (55). Atrogin-1 and MuRF-1 are two members of E3 ubiquitin ligases mastering the ubiquitin-mediated protein degradation (58). Bnip3, an autophagy-related gene, has been reported to regulate CMA in a model of mechanical unloading (59).

**Figure 1:**
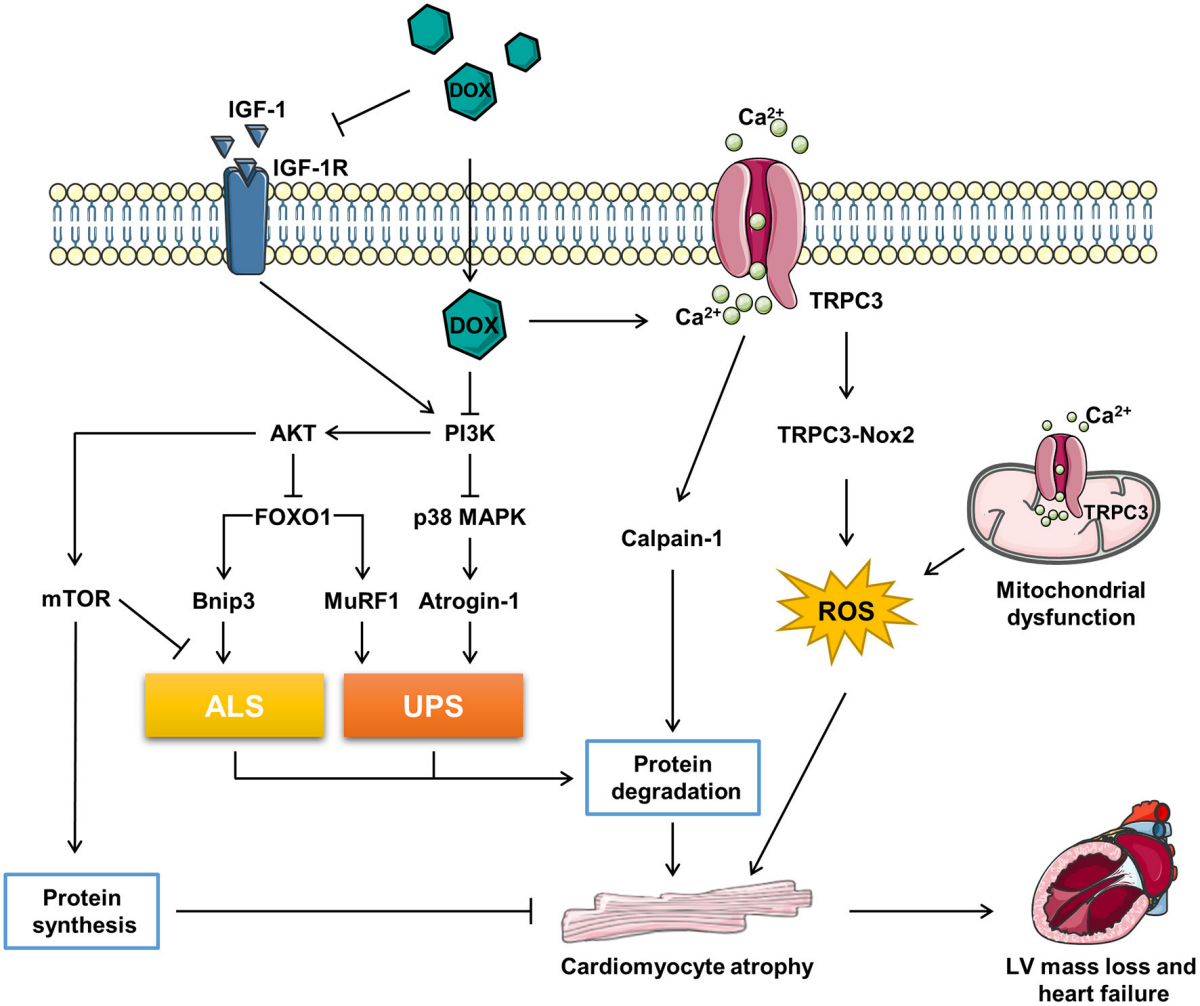
The related molecular mechanism of doxorubicin (DOX)-induced cardiomyocyte atrophy (CMA). ALS, autophagy–lysosome systems; atrogin-1/MAFbx, muscle atrophy gene-1; Bnip3, Bcl-2 19-kDa interacting protein 3; DOX, doxorubicin; FOXO1, forkhead box O1; IGF-1, insulin-like growth factor; IGF-1R, IGF-1 receptor; MuRF1, muscle RING finger 1; mTOR, mammalian target of rapamycin; Nox2, NADPH oxidase 2; PI3K, phosphoinositide 3-kinase; p38 MAPK, p38 mitogen-activated protein kinase; TRPC3, transient receptor potential canonical 3; UPS, ubiquitin–proteasome systems.

It has been reported that high dose (20 mg/kg) of DOX treatment activated FOXO1 phosphorylation at Ser-249 and upregulated nuclear FOXO1 levels, accompanied with the increased expression of its target gene, MuRF1 within 24 h. Pharmacological inhibition of FOXO1 with AS1842856 decreased MuRF1 and prevented DOX-induced CMA and LV mass loss (60). Consistently, Willis et al. reported that DOX treatment resulted into a significant upregulation of MuRF1 and Bnip3, while MuRF1 depletion reversed DOX-induced cardiac atrophy in mice (11). Yamamoto et al. reported that DOX-induced CMA was abrogated by MG-132, a proteasome inhibitor, indicating that the atrophy response is involved in the UPS (61). Wang et al. reported that 3-MA, an autophagy inhibitor, alleviated the DOX-induced CMA *in vitro* and Ghrelin, a multi-functional peptide hormone, attenuated DOX-induced CMA by inhibiting the excessive autophagy (62) (Figure 1).

The expression of FOXO1 and its target genes might be induced by DOX in a time- and dose-dependent manner. Low dose (5 mg/kg) of DOX failed to induce MuRF1 expression at 24 h (60). In addition, the mRNA levels of FOXO1and Atrogin-1 were not upregulated in mice 7 days after injected with 20 mg/kg DOX (11).

In conclusion, DOX triggers catabolic process involving the induction of ALS and UPS *via* activating FOXO1 and its target genes, which contributes to the CMA. However, FOXOs are classified as tumor suppressor genes (63), inhibition of FOXOs may compromise the anti-tumor effect of DOX. Therefore, more precise and comprehensive studies need to be conducted to figure out if FOXOs inhibition is benefit in DIC therapy in patients with cancer (Figure 1).

### Transient Receptor Potential Canonical 3 (TRPC3)-NADPH Oxidase 2 (Nox2) Axis

Transient receptor potential canonical (TRPC) proteins, regulating intracellular Ca^2+^, K^+^, and Na^+^, are involved in a variety of physiological and pathological processes in cardiovascular system (64). It has been reported that TRPC3 is a risk factor deteriorating the pathological cardiac remodeling (65, 66). TRPC3 was upregulated underlying the DOX-induced hypoxia stress, silence of TRPC3 ameliorated DOX-induced CMA (29). NADPH oxidase 2 (Nox2) is a key regulator accounting for the major reactive oxygen species (ROS) generation in response to cardiac injury. Nox2 knock-out mice exhibited ameliorated CMA and improved the cardiac function against accumulative DOX toxicity, which may be associated with the decrease of NADPH oxidase activity and oxidation (67).

Transient receptor potential canonical 3 (TRPC3) protects Nox2 from proteasome-dependent degradation *via* interacting with it at the specific C-terminal sites and promotes its activation by regulating Ca^2+^ entry (65). The functional interaction of TRPC3 and Nox2 is required for DOX-induced CMA, as the supplement of the TRPC3-C terminal fragment peptide, which disrupted the TRPC3-Nox2 complex without affecting the TRPC3 channel activity, attenuated DOX-induced CMA (29). Further, pharmacological inhibition of TRPC3-Nox2 complex by pyrazole-3 (Pyr3) abrogated DOX-induced CMA and ameliorated cardiotoxicity (29).

However, the downstream mechanism of TRPC3-Nox2 in DOX-induced CMA remains poorly known. It was reported that the mitochondrial dysfunction promoted muscle disuse atrophy by increasing oxidation stress, impairing Ca^2+^ handling, and activating associated cellular degradation processes (68, 69). TRPC3 was found to translocate to the mitochondria to mediate mitochondrial Ca^2+^ homeostasis and regulate the mitochondrial function (70). The number of evidence has revealed that the TRPC3-induced ROS emission and mitochondrial dysfunction participate in cardiac remodeling (65, 66, 71). Ca^2+^ overload is one of the major causes of DIC, Chen et al. reported that the upregulation of TRPC3 and TRPC6 contributed to the Ca^2+^ overload in DIC (72). Calmodulin is a ubiquitously expressed calcium binding protein which plays a key role in transducing intracellular calcium signal (73). Trifluoperazine, a strong calmodulin antagonist, was found to alleviate myofibril degeneration and cardiac atrophy induced by DOX (74). Calpains are Ca^2+^-activated neutral cysteine proteases and comprise two major molecules, calpain-1 and calpain-2 (75). Min et al. reported that DOX-induced skeleton and cardiac atrophy requiring the increased mitochondrial emission of ROS and calpain activation (76). Therefore, it can be speculated that DOX might induce CMA through TPRC3-Nox2 axis by disrupting the mitochondrial function, increasing Ca^2+^ entry, and activating the Ca^2+^-associated calpain protein degradation system (Figure 1).

### Phosphoinositide 3-Kinase (PI3K)

#### Insulin-Like Growth Factor 1 (IGF-1) and PI3K

Insulin-like growth factor 1 (IGF-1), a key growth factor controlling both anabolic and catabolic pathways, plays a critical role in modulating the muscle size and function (76). IGF-1 binding to IGF-1 receptor (IGF-1R) leads to increased phosphorylation of insulin receptor substrate-1 (ISR-1), which recruits phosphoinositide 3-kinase (PI3K) and activates downstream the AKT signaling pathway (77). Besides, IGF binding protein (IGFBP) regulated IGF-1 activity by keeping it away from IGF-1R (78). DOX was reported to impair IGF-1R and upregulate IGFBP *via* p53 activation in H9C2 cells (79, 80). Restoration of IGF-1R-PI3K-AKT signaling pathway increased the cell survival ability against DIC (79, 80). Apart from that, exogenous IGF-1 (81) or insulin (82) were reported to alleviated DOX-induced cardiomyocyte apoptosis *via* stimulating PI3K-AKT. Interestingly, Mousa et al. discovered that the co-treatment of human umbilical cord blood mesenchymal stem cells (hUCB-MSCs) and carvedilol alleviated DOX-induced decrease of cardiac muscle fiber diameter, which is accompanied with the elevation of IGF-1, GATA-binding protein 4 (GATA-4), and vascular endothelial growth factor (VEGF) (83). Studies have uncovered that IGF-1 is a pro-hypertrophic inducer in cardiomyocyte (84, 85). Further, exogenous IGF-1 reversed cisplatin-induced skeleton muscle atrophy through inhibiting PI3K-AKT-FOXO mediated UPS (86). Overexpression of IGF-1 was also found to ameliorate cardiac atrophy in spinal muscular atrophy mice (87). However, whether IGF-1/IGF-1R have the potent to rescue CMA induced by DOX remains unknown (Figure 1).

#### PI3K and AKT

The PI3K-AKT signaling pathway plays a vital role in regulating the muscle hypertrophy and atrophy response (77). Studies have revealed that DOX inhibited PI3K-AKT activity both *in vivo* and *in vitro* (79, 88–90). PI3K, a lipid kinase family transducing receptor tyrosine kinase signaling, is aberrantly upregulated in human cancers frequently (91). Though targeting PI3K is effective in cancer therapy, inadvertently increases its side effect on the heart (92). PI3K is a key note in growth factor signaling as well as a modulator in heart muscle mass and contractility (93). Brent et al. found that specific inhibition of PI3Kα by BYL719 decreased the cross-sectional area of cardiomyocyte and induced cardiac atrophy (94). The use of PI3K inhibitor enhanced the anti-tumor effect of chemotherapy drugs, such as DOX (95, 96), while cotreatment of DOX and BYL719 aggravated CMA and cardiotoxicity compared with DOX alone (94). In addition, several studies shed the relationship of PI3K-AKT and cardiac atrophy as well. For example, Chen et al. found that total flavonoids stimulated PI3K-AKT and attenuated DOX-induced HW loss, while inhibition of PI3K-AKT abrogated the protection of total flavonoids against DIC (97). Meeran et al. revealed that nerolidol, a sesquiterpene from the essential oils of aromatic plants, alleviated DOX-induced cardiac atrophy possibly *via* the PI3K-AKT pathway (88). Intriguingly, both upregulation and downregulation of PI3K-AKT triggered by DOX has been reported (98, 99). The discrepancy may be explained by different DIC models and detected time. Interestingly, Cao et al. found that AKT activity was induced by DOX in the beginning, while this was suppressed in the long term (100). In addition, it was reported that PI3Kγ inhibition ameliorated DOX-induced CMA and cardiotoxicity as well as reduced tumor growth (101). Therefore, the role of PI3K-AKT in DIC requires deeper research to clarify and subunit specific inhibition of PI3K might be a promising idea.

Phosphoinositide 3-kinase-AKT activation promotes FOXOs to transport from nucleus to cytoplasm, where FOXOs are sequestered by 14-3-3 proteins and stay inactive (102). Several studies have revealed that the inhibition of PI3K-AKT signaling pathway promoted muscle atrophy *via* FOXOs-mediated activation of UPS (103–105). Moreover, Spurthi et al. reported that toll-like receptor 2 deficiency suppressed PI3K-AKT and activated FOXO1-atrogin-1/MuRF1, which resulted into cardiac atrophy in aging mice (106). Ni et al. found that angiotensin II induced cardiac hypertrophy *via* PI3K-AKT-FOXO pathway (107). Therefore, DOX-induced CMA may be associated with PI3K-AKT-FOXO pathway, which need further exploration. Worth to mention, Yamamoto et al. reported that DOX treatment induced a rapid increase of atrogin-1 mRNA expression *via* activation of p38 mitogen-activated protein kinase (MAPK) pathway without modulating the AKT-FOXO pathway (61).

Mammalian target of rapamycin (mTOR), acts as a serine/threonine kinase, plays an important role in regulating the protein synthesis and modulating autophagy by phosphorylating p70S6K and 4E-BP and Ulk-1, respectively (108, 109). The activity of mTOR regulates the cell growth and organ size (110). The AKT-mTOR axis has been reported to be involved in cardiac hypertrophy during volume overload (111). Further, the PI3K-AKT-mTOR signaling pathway has been found to participate in the DOX-induced skeleton muscle atrophy and cancer cachexia-related cardiac atrophy (112, 113). DOX was reported to impair AKT-mTOR axis by several research (82, 114–117). As reported, β2-agonist formoterol was reported to decrease protein degradation partially through inhibiting PI3K-AKT-mTOR mediated ALS, which prevented the muscle mass loss in fasted mice (118). Apart from that, the activation of PI3K-AKT signaling pathway prevented muscle atrophy *via* mTOR-mediated inhibition of ALS (119, 120). Wang et al. found that ghrelin ameliorated DOX-induced CMA by inhibiting excess autophagy *via* stimulating mTOR (62). Additionally, Hullin et al. revealed that enalapril protected against cardiotoxicity and CMA caused by DOX possibly through activating the PI3K-AKT-mTOR pathway (50). To sum up, DOX might cause CMA *via* inhibiting protein synthesis and activating ALS by suppressing the PI3K-AKT-mTOR pathway (Figure 1).

#### PI3K and p38 MAPK

The p38 MAPK family, which responses to the stress stimuli, plays an important role in cardiac development and function (121). The *in vivo* and *in vitro* evidence has shown that DOX activated the p38 MAPK pathway, which contributed to the DIC (89, 100, 122, 123). McLean et al. reported that suppression of PI3Kα with BYL719 or DOX activated p38 MAPK (94). The stimulation of p38 MAPK is correlative with the muscle wasting. Puigserver et al. found that p38 MAPK activation led to mitochondrial uncoupling and energy expenditure in muscle wasting (124). In addition, Fukawa et al. reported that cancer-secreted inflammatory factors resulted into the excessive fatty acid oxidation and the activation of p38 MAPK, which led to muscle atrophy (125). Several studies have revealed that the activation of p38 MAPK was responsible for DOX-induced CMA. Szeto-Schiller 31 (SS31), an antioxidant peptide, inhibited p38 MAPK phosphorylation and CMA induced by DOX (122). Diosgenin, a steroidal saponin of Dioscorea opposite, alleviated DOX-induced HW and HW/BW ratio reduction possibly *via* suppressing p38 MAPK (123). Further, therapeutic inhibition of p38 MAPK signaling mitigated DOX-induced CMA (94). However, the mechanism that downstream the p38 MAPK in DOX-induced CMA is beyond well established. It was reported that p38 MAPK activation resulted into the upregulation of atrogin-1 and the activation of catabolic process in cancer-induced muscle wasting (126). Pharmacological inhibition of p38 MAPK blunted DOX-induced atrogin-1 upregulation in cardiomyocytes and overexpression of atrogin-1 resulted into CMA (61). Besides, Odeh et al. reported that compromised p38 MAPK activity prevented the denervation-induced muscle atrophy through inhibiting UPS, decreasing oxidation stress, and increased clearance of damaged mitochondria (127). Ding et al. found that Activin A induced skeleton muscle atrophy *via* p38 mediated activation of UPS and autophagy, shown by the upregulation of atrogin-1 and LC3II (128). Therefore, DOX may induce CMA by activating catabolic process though PI3K-p38-atrogin-1 signaling pathway (Figure 1).

## Therapy Strategies

### Exercise

Appropriate exercise has been demonstrated to be beneficial for alleviating the muscle atrophy and improving the muscle strength (129). Wang et al. reported that moderate aerobic exercise decreased DOX exposure in cardiac tissue without altering the microvascular density (130). They found that moderate aerobic exercise during DOX treatment counteracted heart mass loss and cardiac function decline in juvenile tumor-bearing nude mice, while failed to preserve the cardiac function when exercise started after the closure of chemotherapy (130). Gomes-Santos et al. (131) found that aerobic exercise training prevented CMA, ameliorated cardiac atrophy, and attenuated exercise intolerance in mice developed with chronic DIC. While the LVEF reduction and fibrosis were not mitigated by it. Several studies have revealed the molecular mechanism underlying the effect of exercise in ameliorating DOX-induced CMA. Activation of TRPC3-Nox2 pathway contributes to the DOX-induced CMA, it was reported that voluntary exercise downregulated TRPC3 and Nox2 in a posttranslational manner (29). Further, it was reported that exercise upregulated IGF-1 mRNA expression (132) and activated PI3K-AKT impaired by DOX (133). Additionally, Kavazis et al. reported that the short-term endurance exercise training attenuated mRNA expression of some negative regulators of cardiac mass, such as FOXO1, MuRF1, myostatin but not atrogin-1, and Bnip3, which was probably associated with the activation of AMPK/PGC-1α pathway (134).

### Non-Coding RNA (NcRNA)

Non-coding RNA (ncRNA), such as microRNA, small interference RNA (siRNA), long non-coding RNA (lncRNA), and circular RNA (cirRNA), plays an important role in regulating the cardiovascular system (135). Hu et al. reported that DOX treatment resulted into miR-200a downregulation both *in vivo* and *in vitro*, overexpression of miR-200a alleviated DOX-induced cardiac atrophy and cardiac dysfunction *via* nuclear factor (erythroid-derived 2)-like 2 (Nrf2) activation (136). Li et al. (137) also found that DOX caused elevation of miR-451 expression and miR-451 inhibition prevented the whole body wasting and cardiac atrophy and alleviated cardiotoxicity through AMPK signaling pathway in DIC mice. Moreover, Gupta et al. found that miR-212/132, a pro-hypertrophic cluster, ameliorated DOX-induced CMA and improved cardiac function by inhibiting downstream fat storage-inducing transmembrane protein 2 (Fitm2) (138). In addition, they found that Quaking, an RNA-binding protein, exerted cardiac protective effect against DOX-induced CMA and cardiotoxicity *via* mediating cardiac cirRNAs derived from Titin (Ttn), Formin homology 2 domain containing 3 (Fhod3), and Striatin calmodulin-binding protein 3 (Strn3) (139). It seems that interfering with ncRNAs may provide a new strategy in reversing the DOX-induced CMA, however, the related studies remain limited.

### Hormones and Growth Factors

Growing evidence has demonstrated that part of endogenous hormones and growth factors have protective effect in cardiovascular diseases (140–143). Vascular endothelial growth factor-B (VEGF-B), one of the five known members of VEGF that regulate endothelial function (144), has been demonstrated to show potent in promoting coronary arteriogenesis and physiological cardiac hypertrophy (145). Räsänen et al. reported that overexpression of VEGF-B reversed CMA and cardiac mass loss through protecting endothelial in DOX-treated mice without compromising the anti-tumor effect of DOX (146). Li et al. (44) reported that exogenous supplementation of erythropoietin ameliorated DOX-induced CMA and cardiac dysfunction. The same team found that the atrophic response was attenuated by giving granulocyte colony-stimulating factor (G-CSF) in acute DIC mice in their following study (43). Interestingly, Esaki et al. reported that artificial upregulation of hepatocyte growth factor (HGF) at 2 weeks after the establishment of acute DIC model mitigated DOX-induced CMA and cardiac dysfunction (42). The related mechanism underlies the anti-atrophic effect of erythropoietin, G-CSF, and HGF might be similar, which was related to the activation of extracellular signal-regulated kinase (ERK) as well as the restoration of the expression of GATA-4 and its downstream 3 sarcomeric proteins, myosin heavy chain, troponin I, and desmin (42–44). GATA-4, a member of the GATA family of zinc finger transcription factors, is a major transcription factor regulating sarcomeric genes (147). DOX treatment caused a decrease in the level of GATA-4 DNA-binding activity as a result of downregulation of GATA-4 (148), which downregulated the sarcomeric proteins, and resulted into the degeneration of myofibrils in response to DOX.

### Polyphenolic Compounds

The plant-derived polyphenolic compounds exert powerful antioxidant activity and have showed their beneficial effects in cardiovascular disease, such as DIC (149). The polyphenolic compounds can be classified as flavonoids, stilbenes, phenolic acids, and lignans based on the molecular structure (150). Rutin, a polyphenolic flavonoid, prevented DOX-induced cardiac atrophy and dysfunction *via* inhibiting excessive autophagy, reducing apoptosis, and restoring AKT activity (151). Isorhapontigenin, a new derivative of stilbene, alleviated CMA and cardiac atrophy caused by DOX, which is associated with the upregulation of yes-associated protein 1 expression (31). Resveratrol (3,5,4′-trihydroxy-trans-stilbene, RES), a natural polyphenol which can be found mainly in grapes, red wine, soy, and peanuts, has been well studied in DIC protection (152). Earlier, Zhang et al. found that RES prevented DOX-induced HW, BW, HW/BW ratio reduction, and cardiotoxicity *via* sirtuin 1(SIRT1)-p53 pathway (153). Furthermore, Arafa et al. revealed that RES was capable of alleviating cardiac atrophy caused by DOX (154). Several studies have implicated the possible molecular mechanism of the protection of RES on DOX-induced cardiac atrophy. It was reported that RES inhibited DOX-induced catabolic process as indicated by the downregulation of MuRF1 and ubiquitin-specific protease 7 (USP7) *via* increasing the deacetylase activity of SIRT1 in young mice (155). RES was reported to suppress DOX-induced p38 MAPK activation (24, 156) and restore VEGF-B and AKT impaired by DOX (157). Recently, Maayah et al. reported that RES ameliorated DOX-induced cardiac atrophy and cardiotoxicity through inhibiting nucleotide-binding domain-like receptor protein-3 (NLRP3) and systemic inflammation in juvenile mice (25). Interestingly, they found that RES restored DOX-induced deficiency of compensated hypertrophic response to the late-onset hypertension, as indicated by the alleviated CMA and increased heart wall thickness (25). Of note, some polyphenolic compounds have shown the effectiveness against cancer cells both *in vivo* and *in vitro* (158).

### Clinical Drugs

The advantage of clinical drugs is the proved relative safety and the convenience for application. Here, we presented several studies about the protective effect of clinical drugs in DOX-induced CMA. Although the results of clinical study showed that only 11% patients showed complete recovery from DIC receiving conventional HF drugs (10), which may be associated with the underlying mechanism of DIC is cardiac atrophy rather than pathological hypertrophy. Losartan, a clinical used AT1 receptor antagonist, exerted cardioprotective effect against DOX-induced CMA possibly by inhibiting the Nox2 activity (67). Controversial studies about the effect of eplerenone on DIC were reported (50, 159). Enalapril, an angiotensin converting enzyme inhibitor (ACEI), attenuated DOX-induced CMA possibly *via* stimulating the PI3K-AKT-mTOR pathway and maintaining the normal levels of connective tissue growth factor (50). So, it reminds us that is it possible for some specific group population to benefit from the conventional HF drugs in DIC therapy? Oral supplementation of folic acid prevented myofibrils disruption, ameliorated DOX-induced CMA, and improved cardiac function (160). Of note, Durham et al. reported that upregulation of high-density lipoprotein (HDL) by overexpressing apolipoprotein A1 abrogated DOX-induce CMA in mice, which was required for the high-affinity HDL receptor, scavenger receptor class B type 1 (49). This study implicates that a lipid-lowering therapy may be beneficial for DOX-induced CMA.

The phosphodiesterase 5 (PDE5) inhibitors, such as tadalafil, sildenafil, and vardenafil, have been demonstrated to show protection in cardiovascular system (161). Koka et al. revealed that tadalafil, a long-acting selective inhibitor of cGMP-specific PDE5, improved cardiac function, reduced oxidation stress, attenuated apoptosis, and prevented cardiac atrophy in DIC mice (162). Prysyazhna et al. found that tadalafil protected against DOX-induced LV mass loss *via* attenuating protein kinase G Iαoxidation (163). Moreover, Jin et al. reported that tadalafil ameliorated the downregulation of 3 sarcomeric proteins, myosin heavy chain, troponin I, desmin, and alleviated CMA caused by DOX in mice (41). Another PDE5 inhibitor, sildenafil, has been verified to attenuate cardiac dysfunction, apoptosis, mitochondrial damage, and myofibrillar disarray induced by DOX (164). Multiple studies have reported that the administration of PDE5 inhibitors did not affect the anticancer effect but enhanced chemotherapeutic efficacy of DOX in animal tumor models (165–168). However, Poklepovic et al. found that sildenafil was safe, but did not show cardiac protection following DOX treatment in a small randomized clinical trial (169). The effect of sildenafil in DIC will require deeper research to verify. Worth to mention, several studies have shed light into the cardiac protective effect of other PDE inhibitors against DIC. Nishiyama et al. found that ibudilast, a PDE4 inhibitor already used in clinic, exerted cardioprotective effect against DOX-induced CMA by interfering the TRPC3-Nox2 complex without affecting the TRPC3 activity (170). Recently, Chen et al. reported that PDE10A deficiency ameliorated DOX-induced CMA and cardiotoxicity *via* cGMP and cAMP, and PDE10A inhibition antagonized tumor growth (171). Inspiringly, the safety of several PDE10A inhibitors have been demonstrated in phase I clinical trial (171). Zhang et al. revealed that PDE1C deficiency or suppression of ameliorated DOX-induced cardiac atrophy and improved cardiac function *via* adenosine A2 receptor stimulation (172). Cilostazol, a potent PDE3 inhibitor, also alleviated HW loss in DIC (173).

## Discussion

In this review, we pointed out the importance of CMA in DIC and then, summarized recent advances in the molecular mechanism and the promising therapy strategies of DOX-induced CMA. Here, we paid more attention to the studies involving DOX-induced CMA, but not merely cardiac atrophy. Cardiac atrophy is a common finding and a major cause in the DIC. The weight of CMA in cardiac atrophy might be greater than we thought before. In addition, the reversibility of DIC also supports it (174). We are not going to say that we should downgrade the role of cell death yet. Although several studies have reported that little apoptotic effect was found in acute DIC models, the part of cardiomyocyte necrosis was not evaluated (11, 42–44). The apoptotic rate may be underestimated due to the secondary necrosis (175, 176). So, the relative contribution of CMA and cell death in DOX-induced cardiac atrophy is worth to elucidate in the future study. Inhibiting cellular degradation processes and promoting synthesis processes might be the key idea in preventing the DOX-induced CMA. The DOX-induced CMA is a degenerated process, which explains the protective effect of pro-growth therapy, such as exercise and supplementation of growth factors. Pathological hypertrophy is found in multi cardiovascular diseases; however, appropriate hypertrophy can be helpful for alleviating the DOX-induced CMA as proved by Gupta et al. (138). Considering that the cardiac regeneration technology is still far from application in clinic nowadays (177), reversing CMA serves an alternative and promising strategy in DIC therapy.

## Author Contributions

D-SC collected the literature and wrote the manuscript. JY and P-ZY conceived the idea and supervised the manuscript. All authors agree to be accountable for the content of the work. All authors contributed to the article and approved the submitted version.

## Funding

This work was supported by the National Natural Science Foundation of China (Grant Nos. 82070247 and 82000249) and the Guangdong Basic and Applied Basic Research Foundation (Grant No. 2020A1515111028).

## Conflict of Interest

The authors declare that the research was conducted in the absence of any commercial or financial relationships that could be construed as a potential conflict of interest.

## Publisher's Note

All claims expressed in this article are solely those of the authors and do not necessarily represent those of their affiliated organizations, or those of the publisher, the editors and the reviewers. Any product that may be evaluated in this article, or claim that may be made by its manufacturer, is not guaranteed or endorsed by the publisher.
